# Development of Human Monoclonal Antibody for Claudin-3 Overexpressing Carcinoma Targeting

**DOI:** 10.3390/biom10010051

**Published:** 2019-12-28

**Authors:** Hobin Yang, Hayeon Park, Yong Jin Lee, Jun Young Choi, TaeEun Kim, Nirmal Rajasekaran, Saehyung Lee, Kyoung Song, Sungyoul Hong, Joon-Seok Choi, Hyunbo Shim, Young-Deug Kim, Soohyun Hwang, Yoon-La Choi, Young Kee Shin

**Affiliations:** 1 Laboratory of Molecular Pathology and Cancer Genomics, Research Institute of Pharmaceutical Sciences and College of Pharmacy, Seoul National University, Seoul 08826, Korea; dbdyd99@snu.ac.kr (H.Y.); kimte8001@gmail.com (T.K.); nirmalpharma@gmail.com (N.R.); sungyoul@snu.ac.kr (S.H.); 2 ABION Inc., R&D Center, Seoul 08394, Korea; candybk@abionbio.com (H.P.); yongjinl1@abionbio.com (Y.J.L.); jychoi@abionbio.com (J.Y.C.); todnos@gmail.com (Y.-D.K.); 3 Molecular Medicine and Biopharmaceutical Sciences, Graduate School of Convergence Science and Technology, Seoul National University, Seoul 08826, Korea; ronnie85@snu.ac.kr; 4 Center for Companion Diagnostics, LOGONE Bio Convergence Research Foundation, Seoul 08826, Korea; sk17@logonebio.org; 5 College of Pharmacy Daegu Catholic University Hayang-ro 13-13 Gyeongsan-si, Hayang-eup, Gyeongbuk 38430, Korea; joonschoi@naver.com; 6 Department of Life Science, College of Natural Science, Ewha Womans University, Seoul 03760, Korea; hshim@ewha.ac.kr; 7 Department of Pathology and Translational Genomics, Samsung Medical Center, Sungkyunkwan University School of Medicine, Seoul 06351, Korea; juzzsaw@gmail.com (S.H.); yunachoi2468@gmail.com (Y.-L.C.); 8 College of Pharmacy, Bio-MAX, and Research Institute of Pharmaceutical Sciences, Seoul National University, Seoul 08826, Korea

**Keywords:** claudin, tight junction, epithelial tumor, human monoclonal antibody

## Abstract

Most malignant tumors originate from epithelial tissues in which tight junctions mediate cell–cell interactions. Tight junction proteins, especially claudin-3 (CLDN3), are overexpressed in various cancers. Claudin-3 is exposed externally during tumorigenesis making it a potential biomarker and therapeutic target. However, the development of antibodies against specific CLDN proteins is difficult, because CLDNs are four-transmembrane domain proteins with high homology among CLDN family members and species. Here, we developed a human IgG1 monoclonal antibody (h4G3) against CLDN3 through scFv phage display using CLDN3-overexpressing stable cells and CLDN3-embedded lipoparticles as antigens. The h4G3 recognized the native conformation of human and mouse CLDN3 without cross-reactivity to other CLDNs. The binding kinetics of h4G3 demonstrated a sub-nanomolar affinity for CLDN3 expressed on the cell surface. The h4G3 showed antibody-dependent cellular cytotoxicity (ADCC) according to CLDN3 expression levels in various cancer cells by the activation of FcγRIIIa (CD16a). The biodistribution of h4G3 was analyzed by intravenous injection of fluorescence-conjugated h4G3 which showed that it localized to the tumor site in xenograft mice bearing CLDN3-expressing tumors. These results indicate that h4G3 recognizes CLDN3 specifically, suggesting its value for cancer diagnosis, antibody-drug conjugates, and potentially as a chimeric antigen receptor (CAR) for CLDN3-expressing pan-carcinoma.

## 1. Introduction

Malignant tumors, which account for approximately 90% of human cancers, arise from epithelial tissues. Epithelial cells are connected via epithelial sheets that line organ cavities. Tight junctions (TJs) regulate permeability across epithelial sheets and play a role in the development and maintenance of cell polarity [1]. Loss of TJ integrity leads to aberrant cell growth by increasing the influx of growth factor; it promotes the detachment of malignant cells from the primary tumor site by disrupting cell adhesion and polarity, resulting in the formation of distant metastasis [2,3]. Tight junction components are attractive targets, since they are accessible in cancer cells compared to normal cells. In normal epithelia, cells grow parallel to the lumen maintaining their polarity, and the accessibility to the TJ components, located on the apical side of the epithelium, is limited. However, in the initial phase of epithelial tumorigenesis, the orientation of mitotic spindles becomes irregular, leading to out-of-plane division [4]. The misorientation of the cell division axis can change the pattern of the cell–cell adhesion system and cause TJ components to be exposed externally by rotation of the spindle. Hence, TJ components are potential therapeutic targets for cancer treatment or delivery of anticancer agents, specifically into tumor tissues [5,6,7,8].

Claudins (CLDNs) are four-transmembrane domain proteins and components of TJs of epithelial or endothelial cells; they function in paracellular permeability by forming TJ seals that prevent the free movement of lipids, proteins, and solutes between cells [9,10]. Human claudin-3 (hCLDN3) has 220 amino acids (aa) and consist of cytoplasmic N- and C-termini, four transmembrane domains, and two extracellular loops, ECL1 (aa 27–80) and ECL2 (aa 144–159) from the N-terminus [11]. Although the role of CLDN3 in carcinogenesis is unclear and controversial [12,13,14,15,16], high expression of CLDN3 has been reported in various carcinomas including breast, colorectal, gastric, pancreatic, prostate, and ovarian cancer [17,18,19,20]. High CLDN3 expression is correlated with poor prognosis and survival [21,22,23,24], and CLDN3 is considered a pan-carcinoma biomarker.

Because of its expression in various carcinomas, CLDN3 has been investigated as a therapeutic target. Clostridium perfringens enterotoxin (CPE), which cause food poisoning, recognizes ECL2 of CLDN3 and CLDN4 [25,26]. Clostridium perfringens enterotoxin, C-terminal fragment of CPE (C-CPE, a CLDN binding domain without cytotoxicity), and C-CPE fusion protein have been investigated for their therapeutic potential in cancer. Clostridium perfringens enterotoxin shows anti-tumor efficacy in prostate cancer [8], breast cancer [27], and ovarian cancer [28]. C-terminal fragment of CPE increases the efficacy of chemotherapy in ovarian cancer [29]. C-terminal fragment of CPE fusion proteins show cytotoxicity effects in tumors [30,31] and have been investigated as possible drug carriers [32]. Moreover, C-CPE has been used as an optical imaging agent and as a potential carrier to specifically deliver therapeutic drugs in ovarian cancer [33,34]. These approaches using CPE provide proof of concept for cancer diagnosis and treatment targeting CLDN3 [35,36]. However, CPE can also bind to CLDN5, CLDN6, CLDN7, CLDN9, and CLDN14 [26]. C-terminal fragment of CPE induced an immune response in mice [37], and C-CPE-fused toxin led to hepatic injury [38]. Therefore, it is necessary to develop therapeutics targeting CLDN3 with high specificity, low immunogenicity, and low toxicity.

Antibodies are highly specific antigen-targeting molecules with low immunogenicity and toxicity [39]. The bivalent property of antibody displays an avidity effect which is the accumulated strength of multiple affinities and is commonly referred to as a functional affinity. The avidity plays critical roles in penetration, catabolism, specificity, and efficacy [40]. For these reasons, many therapeutic antibodies have been developed, and over 80 therapeutic antibodies have been approved by the FDA [41]. Antibodies kill tumor cells directly through receptor blockade, antibody-dependent cellular cytotoxicity (ADCC) or complement-dependent cytotoxicity (CDC) [42,43]. In addition, the precision targeting moiety of antibody allows specific and selective delivery of therapeutic agents including cytotoxic agents [44], radioisotopes [45], toxins [46] or photosensitive agents [47]. Diagnostic probes, such as fluorophores, radioisotopes, computed tomography (CT) contrast agents or paramagnetic particles, are also available for antibody-based imaging in a systemic and non-invasive manner for early cancer diagnosis, detecting cancer lesions, and monitoring the prognosis of treatment [48,49].

It is difficult to produce antibodies against CLDNs because extracellular loops are so short that they show low immunogenicity; in addition, they have high sequence homology among humans, mice, and rats [50]. Moreover, there are limitations associated with mimicking the four-transmembrane structure using recombinant proteins. In this study, we generated a human immunoglobulin G1 (IgG1) monoclonal antibody (h4G3) against CLDN3 from a human single-chain variable fragment (scFv) phage library [51] using CLDN3-expressing CHO-K1 cells and CLDN3-embedded lipoparticles as antigens and confirmed its specific binding to human and mouse CLDN3 without affinity to other CLDN family members. We demonstrated the ADCC activity of h4G3 in many types of cancer cell lines according to CLDN3 expression levels. Finally, we visualized that h4G3 specifically recognized CLDN3-expressing tumors rather than normal organs in mouse xenograft models.

## 2. Materials and Methods

### 2.1. Cell Lines and Cell Culture

Human breast cancer (T47D, MCF-7, and HCC202), human ovarian cancer (OVCAR-3, Caov-3, and TOV-112D), human colon cancer (T84, NCI-H508, and SW1116), human gastric cancer (SNU-216 and Hs746T), human liver cancer (NCI-H684), and human pancreatic cancer (AsPC-1) cell lines were used as target cancer cells. The CHO-K1 (Chinese hamster ovary), L cells (mouse fibroblast), HEK293 (human embryonal kidney), and NK-92MI (human lymphoblast) cells were used for preparing the transfectants. All cell lines were purchased from Korean Cell Line Bank (KCLB; Seoul, Korea), except TOV-112D, T84, CHO-K1, L cells, and NK-92MI cells, which were purchased from the American Type Culture Collection (ATCC; Manassas, VA, USA). The Caov-3, Hs578T, and NCI-H684 cells were cultured in DMEM (HyClone, Logan, UT, USA) and supplemented with 10% fetal bovine serum (FBS; HyClone), 100 units/mL penicillin, and 100 μg/mL streptomycin. The TOV-112D cells were maintained in a 1:1 mixture of Media199/MCDB medium (HyClone) containing 15% FBS, 100 units/mL penicillin, and 100 μg/mL streptomycin. The T84 cells were cultured in DMEM/F12 (Gibco, Carlsbad, CA, USA) containing 5% FBS, 100 units/mL penicillin, and 100 μg/mL streptomycin. The NK-92MI cells were maintained in MEM alpha (Gibco) containing 20% FBS, 100 units/mL penicillin, and 100 μg/mL streptomycin. All other cell lines were cultured in RPMI-1640 (HyClone) supplemented with 10% FBS, 100 units/mL penicillin, and 100 μg/mL streptomycin. All cells were incubated at 37 °C in humidified 5% CO_2_ atmosphere.

### 2.2. Establishment of Stable CLDN Transfectants

The cDNAs for CLDN1, 3, 4, 5, 6, 8, 9, and 17 were cloned into pcDNA3.1(+) (Invitrogen, Carlsbad, CA, USA). The CLDN3 expression construct was then transfected into CHO-K1, L cells, HEK293, and TOV-112D cells using FuGENE HD transfection reagent (Promega, Madison, WI, USA); other CLDN expression constructs were transfected into HEK293 cells. After transfection, G418-resistant cells were selected and isolated using a clonal cylinder. To generate chimeric ECL fusion CLDN1/CLDN3 transfectants, cDNA fragments encoding aa 1~104 of CLDN1 fused with aa 104~220 of CLDN3 and aa 1~103 of CLDN3 fused with aa 105~211 of CLDN1 were transfected into HEK293 cells and subjected to the procedure described above.

### 2.3. Establishment of NK-92MI-CD16a Cell Line

A high affinity variant (158V) of FcγRIIIa (CD16a) cDNA was cloned into pcDNA3.1(+) (Invitrogen). The NK-92MI cells were transfected with the CD16a plasmid by electroporation and selected with G418. After selection, CD16a-expressing cells were isolated using BD FACSAria^TM^ III (BD Biosciences, San Jose, CA, USA) at high-expression populations.

### 2.4. Production of Human Monoclonal Antibody (mAb)

The Freedom pCHO 1.0 vector (Gibco), including heavy and light chains of h4G3, was transfected into Freedom CHO-S cells (Gibco) in accordance with the manufacturer’s instructions to establish stable h4G3-expressing CHO-S cells. This transfectant was fed 4 g/L glucose on day 3 and 5, and 6 g/L glucose on day 7, and incubated for up to 2 weeks. The culture supernatant was loaded onto MabSelect SuRe Protein A resin (GE Healthcare, Piscataway, NJ, USA). The resin was washed with five column volumes (CVs) of 35 mM sodium phosphate and 500 mM NaCl (pH 7.2). The bound antibody was eluted with five CVs of 0.1 M sodium citrate (pH 3.6) and neutralized by 1 M Tris-HCl (pH 8.0). The buffer exchange and concentration were achieved using an Amicon Ultra-15 (Merck Millipore, Billerica, MA, USA). The purified antibody was quantified using the Cedex Bio Analyzer (Roche, Indianapolis, IN, USA), and the intact form of the antibody was analyzed using SDS-PAGE under reducing and non-reducing conditions.

### 2.5. Flow Cytometry Analysis

To analyze h4G3 binding to CLDN, cells were detached with enzyme-free, PBS-based cell dissociation buffer (Gibco). A total of 2.5 × 10^5^ cells were incubated with human IgG (10 μg/mL) (Jackson Immunoresearch Laboratories, West Grove, PA, USA) or h4G3 (10 μg/mL) in PBS containing 1% FBS for 1 h on ice. The cells were then washed three times with PBS containing 1% FBS and incubated with goat anti-human IgG-FITC (Jackson Immunoresearch Laboratories) (1:100 dilution) for 1 h on ice. Stained cells were washed three times and analyzed using a BD FACSCalibur system equipped with the Cell Quest Pro software (BD Biosciences).

### 2.6. Western Blotting and Immunoprecipitation

For Western blotting, cells were lysed in RIPA lysis buffer (150 mM sodium chloride, 1% Triton X-100, 1% sodium deoxycholate, 0.1% SDS, 50 mM Tric-HCl, pH 7.4, 2 mM EDTA) including protease inhibitor cocktail (Roche). The supernatant was collected by centrifugation at 14,000 rpm for 15 min at 4 °C, and protein concentration was determined using the BCA protein assay kit (Thermo Fisher Scientific, Waltham, MA, USA). Lysed proteins were resolved by 15% SDS-PAGE and transferred to a polyvinyl difluoride (PVDF) membrane (Bio-Rad Laboratories, Hercules, CA, USA). The membrane was then blotted with anti-CLDN1 (Santa Cruz Biotechnology, Santa Cruz, CA, USA), anti-CLDN3 antibody (Abcam, Cambridge, UK) or β-actin (Santa Cruz Biotechnology). For immunoprecipitation under non-denaturing conditions, cells were resuspended in 600 μL PBS containing a protease inhibitor cocktail (Roche) and sonicated for 10 cycles of 2 s pulses on and 5 s pulses off using a Branson Digital Sonifier 450 (Branson Ultrasonics, Danbury, CT, USA). After quantifying the lysed proteins using the BCA protein assay kit, 1 mg of proteins was incubated with 1 μg human IgG (Jackson Immunoresearch Laboratories) or 1 μg h4G3 for 1 h at 4 °C with rotation. The antibody-mixed lysate was incubated with 50 μL of Protein A-Agarose (Roche) for 1 h at 4 °C with rotation. The precipitated beads were washed three times with PBS, and the precipitated proteins were analyzed by Western blotting. 

### 2.7. Immunofluorescence

For immunofluorescence, hCLDN3/TOV-112D, TOV-112D, OVCAR-3, and Caov-3 cells were seeded on a 4 well cell culture slide, grown to 80% confluency and treated with 5 μg/mL of human IgG (Jackson Immunoresearch Laboratories) or h4G3 for 1 h at 4 °C. The cell culture slide was washed with PBS, and cells were fixed by 4% formaldehyde for 15 min. Cells were washed with PBS, blocked with PBS containing 5% BSA for 1 h, and incubated with goat anti-human IgG-FITC (Jackson Immunoresearch Laboratories) (1:200 dilution) for 1 h. Cells were washed with PBS, stained with Hoechst 33342 (Invitrogen) for staining nuclei, and mounted using Fluoromount Aqueous Mounting Medium (Sigma–Aldrich, St Louis, MO, USA). Images were taken with the LSM 700 ZEISS laser scanning confocal microscope (Carl Zeiss, Jena, Germany). Data were processed using ZEN confocal software (Carl Zeiss). 

### 2.8. Cell-Based Affinity Kinetics

The binding kinetics against CLDN3 on the cell surface were measured using LigandTracer Green (Ridgeview Instruments AB, Vänge, Sweden). The hCLDN3/HEK293, hCLDN3/TOV-112D, and mCLDN3/HEK293 cells, as positive cells, and HEK293 and TOV-112D cells, as negative cells, were seeded on a limited area of 100 mm culture dish at a density of 3 × 10^5^ cells/mL in 500 μL culture medium, and after 6 h, 10 mL growth medium was added to culture dish. Cells were incubated overnight, and 3 mL of the medium was changed before the experiment. The h4G3 was labeled with DyLight dye 488 using DyLight Antibody Labeling Kits (Thermo Fisher Scientific) following the manufacturer’s instructions. The cell culture dish was clamped onto the device and the fluorescence baseline was recorded. Each time the respective fluorescence reached equilibrium, Dylight dye 488-labeled h4G3 was added stepwise to a final concentration of 3 nM and 9 nM for hCLDN3 cell lines, and 30 nM and 90 nM for mCLDN3 cell lines. In the dissociation phase, the remaining medium was removed, and 3 mL fresh medium was added to the culture dish. All measurements were performed using a 15 s detection time and 4 s detection delay. Recorded data were analyzed by TraceDrawer (Ridgeview Instruments AB).

### 2.9. In Vitro Antibody-Dependent Cellular Cytotoxicity (ADCC) 

The ADCC assay was performed in various cell lines. Target cells were seeded on 96 well plates at a density of 2 × 10^4^ cells/well and incubated overnight. In the following steps, RPMI 1640 with 5% FBS was used for preparing materials. The NK-92MI-CD16a cells, which stably express CD16a, were used as effector cells. The target cells were incubated with human IgG (Jackson Immunoresearch Laboratories) or h4G3 at a final concentration of up to 10 μg/mL and 8 × 10^4^ effector cells in a CO_2_ incubator for 4 h at 37 °C (target cells/effector cells ratio was 1/4). Target cell lysis was measured by detecting the release of lactate dehydrogenase (LDH) using CytoTox 96^®^ Non-Radioactive Cytotoxicity Assay according to the manufacturer’s instructions. The absorbance of the plates was analyzed on a Spark^TM^ 10M microplate reader (Tecan, Männedorf, Switzerland) at 490 nm. For data analysis, the percentage of specific ADCC was calculated as follows:

$$\%\;\mathrm{Cytotoxicity}=\frac{\mathrm{Experimental}-\mathrm{Effector}\;\mathrm{Spontaneous}-\mathrm{Target}\;\mathrm{Spontaneous}}{\mathrm{Target}\;\mathrm{Maximum}-\mathrm{Target}\;\mathrm{Spontaneous}}\times 100$$

The dose–response curve and EC_50_ values were estimated using GraphPad Prism 7 (GraphPad Software, San Diego, CA, USA).

### 2.10. Biodistribution in a Nude Mouse Xenograft Tumor Model

Human IgG (Jackson Immunoresearch Laboratories) and h4G3 were labeled with fluorescence dye CF750 using VivoBrite Rapid Antibody Labeling Kit (Biotium Inc., Hayward, CA, USA) in accordance with manufacturer’s instructions. To generate xenograft, 5 × 10^6^ OVCAR-3 cells in 100 μL PBS were injected subcutaneously into the right flank of 6 week old female athymic nude mice (Orient Bio, Seongnam, Gyeonggi, Korea). For the T47D xenograft model, 1 × 10^7^ T47D cells in 100 μL PBS were injected subcutaneously into athymic nude mice planted with 17β-estradiol pellets (Innovative Research of America, Sarasota, FL, USA). Tumor-bearing mice were treated intravenously with CF750-labeled antibodies as 100 μg/100 μL PBS. After 6, 24, 48, 72, and 96 h, the mice were anesthetized with Terrell^TM^ isoflurane (Piramal Critical Care Inc., Bethlehem, PA, USA) and placed in the IVIS Spectrum CT (Perkin Elmer, Waltham, MA, USA) to visualize CF750-labeled antibodies. The fluorescence was detected using an excitation filter (710 nm) and emission filter (780 nm). At the final time point, the mice were sacrificed and the liver, kidney, lung, spleen, intestine, and tumor were excised. The organs from each mouse were placed in 100 mm Petri dishes and imaged using IVIS Spectrum CT (Perkin Elmer). The intensity of fluorescence was analyzed using Live Imaging software (Perkin Elmer), and the average fluorescence intensity from organs was calculated by creating a region of interest over each organ. All animal experiments were approved by the Institutional Animal Care and Use Committee (IACUC) of Seoul National University (SNU-190216-1).

### 2.11. Statistical Analysis

The significance of differences among groups was evaluated using two-way analysis of variance (ANOVA). Data were analyzed with GraphPad Prism 7 (GraphPad Software) and *p* < 0.05 was considered statistically significant.

## 3. Results

### 3.1. Generation of a Human mAb Against Human CLDN3

To create a monoclonal antibody (mAb) that recognized CLDN3, we isolated the anti-CLDN3 scFv by phage display using human CLDN3-expressing CHO-K1 cells (hCLDN3/CHO-K1) and human CLDN3-embedded lipoparticles as antigens. The scFv selection was monitored by measuring output-to-input ratios (Supplementary Materials Figure S1A) and by ELISA (Figure S1B) which showed the enrichment of scFv against CLDN3. Among 190 selected scFv clones from hCLDN3/CHO-K1 cells panning, a 4G3 clone that showed highly specific binding to CLDN3 by flow cytometry was selected (Figure S2A). In hCLDN3-embedded lipoparticle panning, 165 of the 190 clones were selected by lipoparticle-based ELISA, and the sequencing results confirmed that all clones were identical to the 4G3 clone. The 4G3 scFv clone was converted to human IgG1 (h4G3) and purified using protein A affinity chromatography. The integrity of h4G3 was analyzed by SDS-PAGE which detected the correct size of the IgG heavy and light chains and the full IgG at 50 kDa, 25 kDa, and 150 kD, respectively (Figure S2B). 

**Figure 1:**
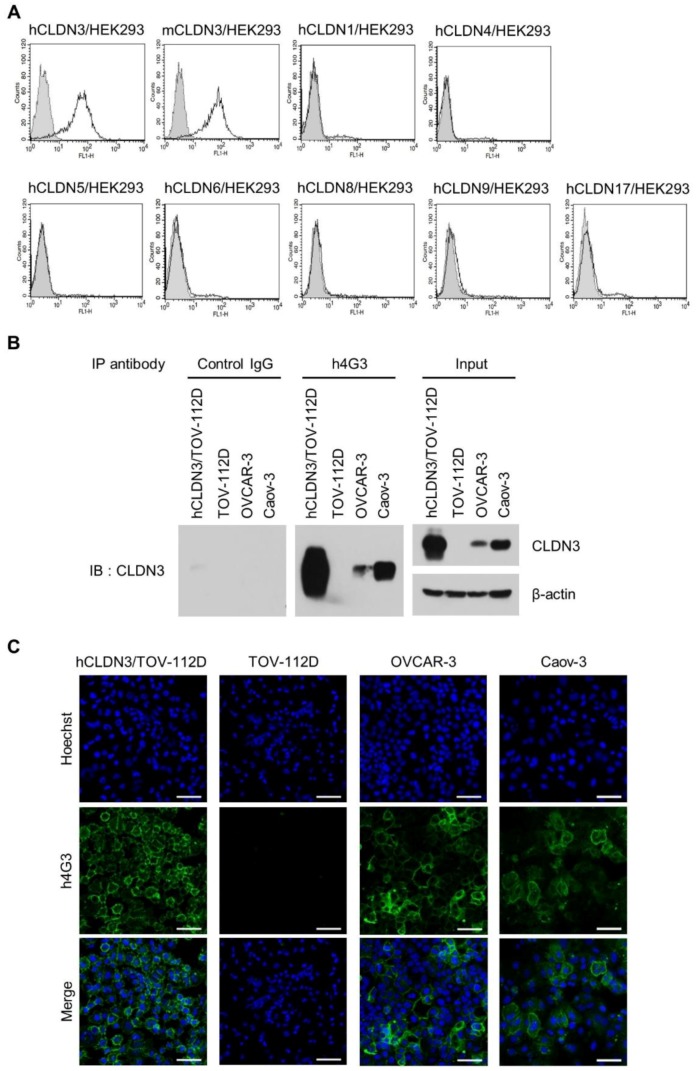
Specificity and conformational structure recognition of h4G3 against claudin-3 (CLDN3). (**A**) Stable CLDN-expressing HEK293 cells were stained with h4G3 and detected by flow cytometry. The gray closed dotted and open solid histograms represent control human immunoglobulin G (IgG)- and h4G3-treated cells, respectively. hCLDN, human CLDN; mCLDN, mouse CLDN. (**B**) The cell lysates were prepared using a probe sonicator in PBS buffer and precipitated with h4G3 or control human IgG. The precipitates were analyzed by Western blotting with anti-CLDN3 antibody. (**C**) hCLDN3/TOV-112D, TOV-112D, OVCAR-3, and Caov-3 cells were incubated with h4G3 for 1 h at 4 °C, fixed, and stained with fluorescein isothiocyanate (FITC)-conjugated goat anti-human IgG. Fluorescence was observed by confocal microscopy. The green and blue signals represent h4G3 and nuclei, respectively. Scale bar = 50 μm.

The CLDN family comprises 26 members in humans [52] with similar structures that form four-transmembrane domain proteins [11]. To verify the specificity of h4G3 for CLDN3 without cross-reactivity to other CLDN family members, hCLDNs/HEK293 cells stably expressing CLDN3, 4, 5, 6, 8, 9, and 17, which are the closest members phylogenetically [11], and CLDN1, which is the canonical CLDN, were generated (Figure S3). The h4G3 bound only to CLDN3 among the stable CLDN transfectants and also bound to mouse CLDN3 (mCLDN3) in mCLDN3/HEK293 cells (Figure 1A). 

In order to verify the recognition in cancer cells, the expression of CLDN3 was confirmed (Figure S4A), and h4G3 binding on the cell surface in various cancer cell lines was observed according to CLDN3 expression (Figure S4B). Because of the structural complexity of CLDN3, the h4G3 did not bind to the recombinant CLDN3 protein or to CLDN3 under denaturing conditions (data not shown). However, h4G3 specifically precipitated CLDN3 from cell lysates prepared under non-denaturing conditions (Figure 1B). Attachment of the h4G3 to the membrane of CLDN3-expressing cancer cells was observed when it was treated to the cells before fixation (Figure 1C). In CLDN3-negative cell lines, the signal of h4G3 was comparable to that of the control IgG, indicating the lack of non-specific binding of the h4G3 to the cell membrane. Taken together, these findings confirmed the successful isolation of the scFv clone (4G3) and the generation of a human mAb (h4G3) that recognizes the conformational structure of both hCLDN3 and mCLDN3 without cross-reactivity to other CLDNs.

### 3.2. h4G3 Recognizes the ECL2 Domain of CLDN3

**Figure 2:**
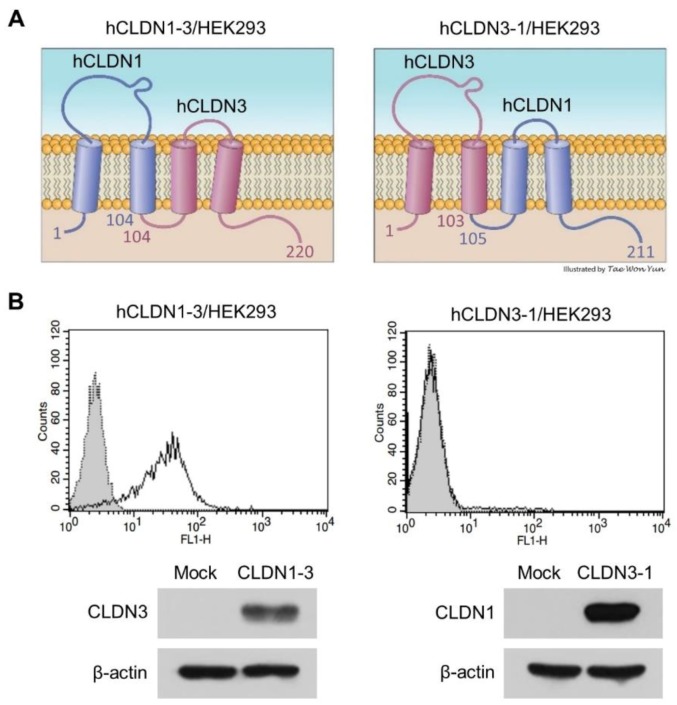
Binding of h4G3 to the second extracellular loop of CLDN3. (**A**) Structure of extracellular domain fusion CLDNs. hCLDN1-3 includes aa 1~104 of CLDN1 and aa 104~220 of CLDN3, whereas hCLDN3-1 consists of aa 1~103 of CLDN3 and aa 105~211 of CLDN1. (**B**) Control human IgG (gray closed dotted histogram) and h4G3 (open solid histogram) were added to hCLDN1-3/HEK293 and hCLDN3-1/HEK293 cells, and the bound antibodies were detected by flow cytometry using FITC-conjugated goat anti-human IgG. The protein expression of transfected fusion genes was detected by immunoblotting with anti-CLDN1 or anti-CLDN3 antibodies, which recognized the C-terminus cytoplasmic domain of the respective CLDN1 and CLDN3.

Binding of the h4G3 to CLDN3 was further analyzed by constructing two chimeric CLDNs as fusion genes between *CLDN1* and *CLDN3* according to a CPE binding study [25]. The hCLDN1-3 contained ECL1 of CLDN1 and ECL2 of CLDN3 (aa 1~104 of CLDN1 and aa 104~220 of CLDN3), and hCLDN3-1 contained ECL2 of CLDN1 and ECL1 of CLDN3 (aa 1~103 of CLDN3 and aa 105~211 of CLDN1) (Figure 2A). We established HEK293 cells stably expressing hCLDN1-3 or hCLDN3-1 and designated them as hCLDN1-3/HEK293 and hCLDN3-1/HEK293, respectively. The expression of chimeric fusion proteins was confirmed by Western blotting using commercial antibodies detecting the C-terminus of each CLDN1 and CLDN3; the anti-CLDN3 antibody recognized hCLDN1-3, and the anti-CLDN1 antibody recognized hCLDN3-1. After exposure to hCLDN1-3/HEK293 cells and hCLDN3-1/HEK293 cells, h4G3 showed binding to hCLDN1-3/HEK293 cells (Figure 2B). These results suggest that h4G3 recognizes the conformational structure of the ECL2 domain of CLDN3 on the cell membrane and binds to mCLDN3 because there is only 1 aa difference in ECL2 between hCLDN3 (150V) and mCLDN3 (150L).

### 3.3. Binding Kinetics of h4G3 to CLDN3 on the Cell Membrane

**Figure 3:**
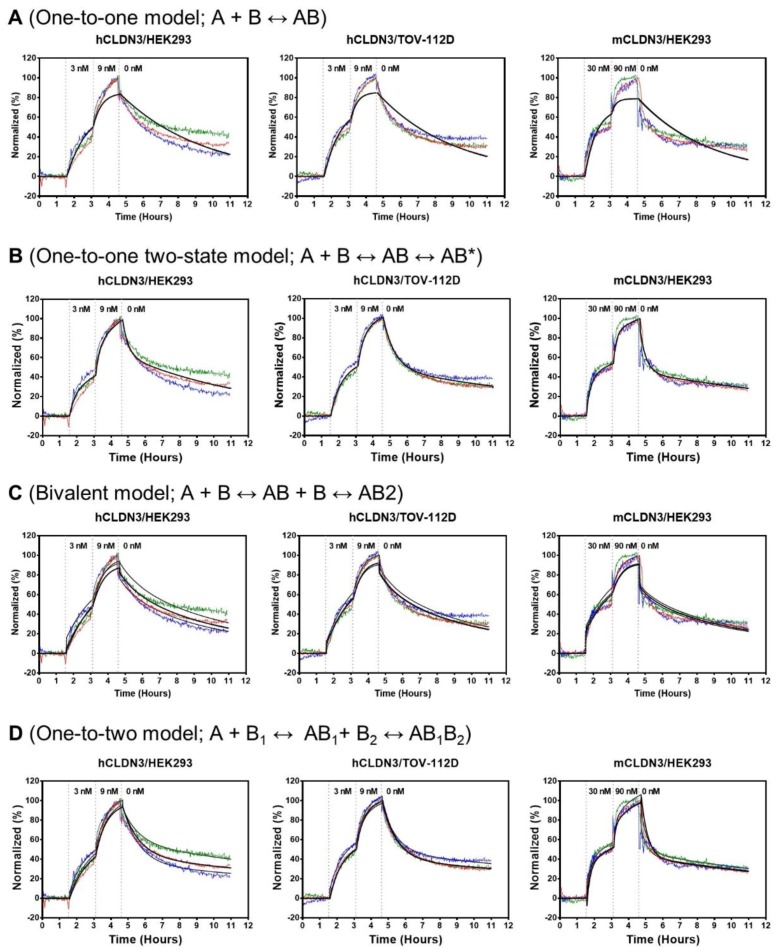
Kinetic traces for FITC-labeled h4G3 binding to human and mouse CLDN3 on the cell membrane. FITC-labeled h4G3 was added to the cells in a stepwise increase to a final concentration of 3 nM (t = 1.5 h) and 9 nM (t = 3 h) for hCLDN3 and 30 nM (t = 1.5 h) and 90 nM (t = 3 h) for mCLDN3. The dissociation phase was achieved by replacement with fresh medium (t = 4.5 h). The signal intensity was calculated by subtracting the value of reference cells from that of positive cells (*n* = 3). Fluorescent signals were normalized to 0% at baseline and 100% at the end of second ligand incubation for visual comparison. Binding curves obtained from three independent experiments are shown in red, green, and blue. “One-to-one” fitting curves (**A**), “one-to-one two-state” fitting curves (**B**), “bivalent” fitting curves (**C**), and “one-to-two” fitting curves (**D**) are shown in black.

Next, we evaluated the binding kinetics of h4G3 to CLDN3 on the cell surface using LigandTracer Green (Ridgeview Instruments AB) which allows measuring real-time binding kinetics on living cells. The hCLDN3/HEK293, hCLDN3/TOV-112D, and mCLDN3/HEK293 cells were used as target cells, whereas HEK293 and TOV-112D cells served as reference cells. After achieving a stable baseline, FITC-labeled h4G3 was sequentially added to a final concentration of 3 nM and 9 nM for hCLDN3. Similarly, for mCLDN3, 30 nM and 90 nM of FITC-labeled h4G3 was added. The dissociation phase was recorded after replacing the incubation solution with fresh medium. The fluorescence signal was higher in hCLDN3-expressing target cells than in reference cells (Figure S5A,B). The difference in the binding intensity between mCLDN3 target cells and reference cells was smaller than that observed for hCLDN3 despite a greater amount of antibody used (Figure S5C). Western blotting using anti-CLDN3 antibody, which recognizes both hCLDN3 and mCLDN3, showed that hCLDN3 and mCLDN3 were expressed at similar levels in the different cell lines after normalizing the protein level to that of β-actin (Figure S5D). The signal intensity of target cells was subtracted with that of each of the reference cells, normalized, and fitted by different types of fitting models. The “one-to-one” model showed poor fitting curves since it is a simple model that cannot represent the complex binding (Figure 3A). Fitting a “one-to-one two-state” model to the binding curves demonstrated an affinity of 4.03 nM in hCLDN3/HEK293 cells, 2.35 nM in hCLDN3/TOV-112D cells, and 20.4 nM in mCLDN3/HEK293 cells, respectively (Figure 3B and Table 1). The “one-to-one two-state” fitting model, which accounts for the second process contribution to the interaction, corresponded better to the measured curve than the simpler “one-to-one” model, because the binding of an antibody to targets on living cells is more complicated than it seems [53]. Since antibodies have bivalent binding which generates avidity, the binding sensorgrams were analyzed with more heterogeneous fitting model. The “bivalent” model also showed poor fitting curve since it may be not very helpful in sorting out the interaction events when the binding of the second antibody arm is a significantly faster process than the binding of the antibody to target with the first arm (Figure 3C). Instead, fitting a “one-to-two” model to the binding curves showed two affinity values; first weak binding affinity and second strong binding affinity due to the slow dissociation by avidity. In the hCLDN3/HEK293, hCLDN3/TOV-112D, and mCLDN3/HEK293 cells, each affinity was evaluated as the first binding affinity (K_D1_ = 8.33, 5.15, and 30.1 nM) and the second binding affinity (K_D2_ = 0.504, 0.434, and 7.54 nM), respectively (Figure 3D and Table 2). The K_D_ values from “one-to-one two-state” model lay in between the K_D1_ and K_D2_ values from “one-to-two” model. The binding affinity of the FITC-labeled h4G3 used in the binding kinetics assay was comparable to that of unlabeled h4G3 in CLDN3-expressing cells (Figure S6A). These data indicate that h4G3 binds hCLDN3 on the cell membrane with typical antibody affinity and shows a lower affinity for mCLDN3.

### 3.4. Evaluation of the Anti-Tumor Activity of h4G3

**Figure 4:**
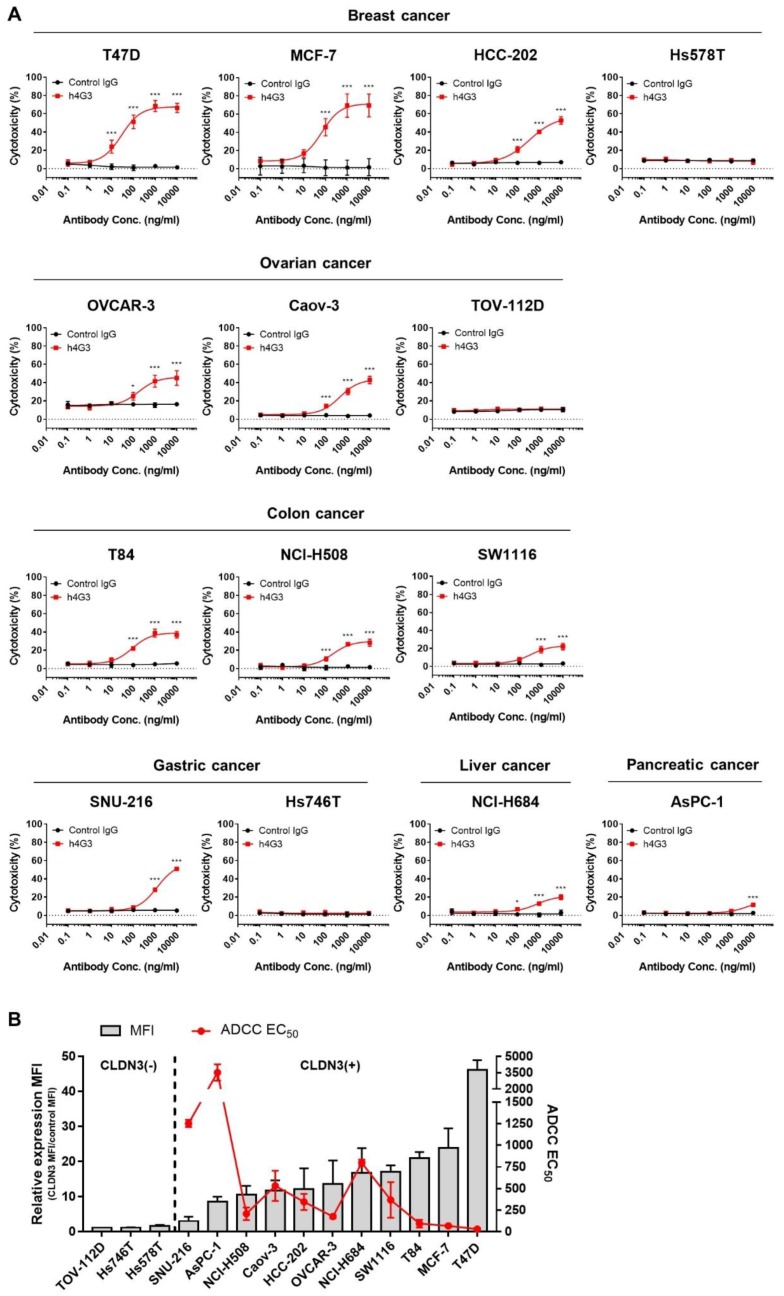
In vitro antibody-dependent cellular cytotoxicity (ADCC) activity of h4G3 in a CLDN3 expression-dependent manner in various types of cancer cell lines. NK-92MI-CD16a cells were used as effector cells. (**A**) Cancer cell lines were incubated with effector cells at a target:effector ratio of 1:4 and various concentration of h4G3 for 4 h at 37 °C. Cytotoxicity (%) was determined by measuring released lactate dehydrogenase (LDH) and normalizing to a maximum LDH release in the presence of Triton X-100 (100% cell lysis). (**B**) Mean fluorescence intensity (MFI) ratio was calculated by dividing the h4G3 MFI by the control IgG MFI, as determined by flow cytometry. ADCC EC_50_ values were calculated by non-linear regression using GraphPad 7.0. The relative CLDN3 MFI expression (bar, left *y*-axis) and the ADCC EC_50_ (red line, right *y*-axis) in each cancer cell line are shown. Data represent the mean ± SD (*n* = 3). * *p* < 0.05, *** *p* < 0.001 versus control IgG.

Most therapeutic antibodies show immune-mediated effector functions such as ADCC and CDC. The ADCC is mediated by activation of the FcγRIIIa (CD16a) on natural killer (NK) cells [54]. Since NK-92MI cells are CD16a-deficient [55], we generated a high-affinity variant (158V) of CD16a-expressing NK-92MI cells (NK-92MI-CD16a) by stable transfection (Figure S7A,B). The ADCC of h4G3 against cancer cells was assessed using NK-92MI-CD16a cells as effector cells, and various types of cancer cells were incubated with the effector cells at a ratio of 1:4 (target cell:effector cell) for 4 h. The cytotoxicity was determined by measuring the activity of LDH released from apoptotic cells by ADCC. In CLDN3-positive cancer cells, h4G3 showed dose-dependent ADCC according to the level of CLDN3 expression through NK-92MI-CD16a cells, and there was no ADCC in CLDN3-negative cancer cells (Figure 4A). The ADCC EC_50_ of h4G3 was correlated with mean fluorescence intensity (MFI) of CLDN3 expression in various cancer cell lines, except for a few cell lines such as AsPC-1 and NCI-H684 (Figure 4B). On the other hand, h4G3 did not have any direct cytotoxicity and CDC activity in CLDN3-positive cell lines (Figure S8A,B). These data demonstrate that the cytotoxic activity of h4G3 is mediated by ADCC according to the expression of CLDN3 and independently of cancer type. 

### 3.5. Tumor Targeting and Accumulation of h4G3 in Xenograft Models

**Figure 5:**
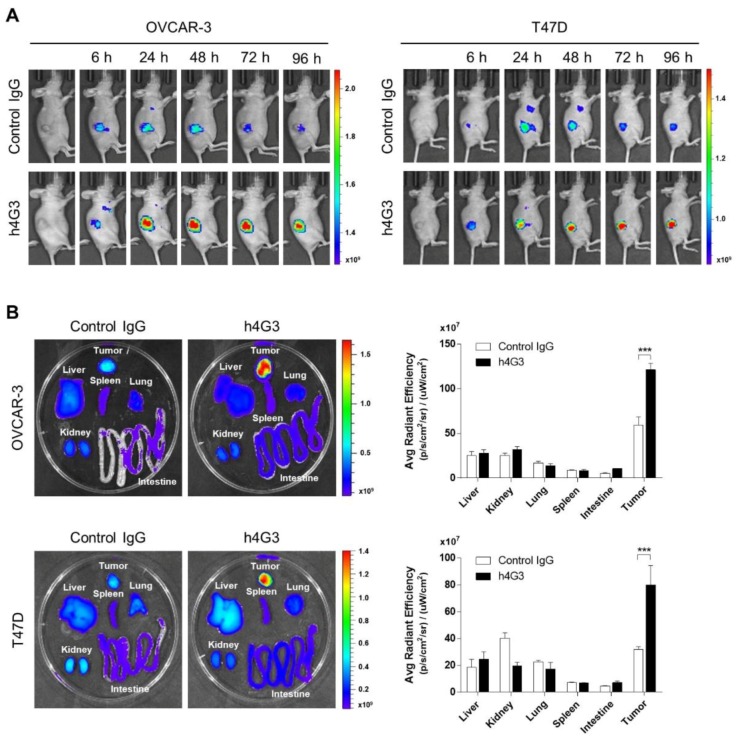
Biodistribution of h4G3 in mice bearing xenograft tumor cells. (**A**) OVCAR-3 and T47D cells were transplanted subcutaneously into athymic nude mice. CF750–control human IgG and CF750–h4G3 were intravenously injected at a dose of 100 μg/mouse, and the fluorescence intensity was monitored at 6, 24, 48, 72, and 96 h. (**B**) Tissues from OVCAR-3 xenograft mice and T47D xenograft mice were isolated at 96 h post-injection, and the ex vivo fluorescence images were observed. The ex vivo fluorescence images from each tissue were quantified as the average radiant efficiency. Data represent the mean ± SEM (*n* = 3), *** *p* < 0.001 versus control human IgG.

To investigate whether h4G3 recognizes CLDN3-overexpressing tumors in vivo, we generated subcutaneous OVCAR-3 and T47D xenograft models. The h4G3 was conjugated to CF750 fluorescent dye, which is suitable for in vivo imaging because it has a low absorption spectrum for biological molecules at the detection range. The binding affinity of CF750-conjugated h4G3 to CLDN3-expressing cells was comparable to that of the h4G3 (Figure S6B). The CF750-conjugated h4G3 was injected into the xenograft mouse models at a dose of 100 μg per mouse, and the distribution signals were monitored in a time-dependent manner. Although a small amount of control IgG was also distributed in the tumor because of the enhanced permeability and retention (EPR) effect, h4G3 accumulated remarkably in the tumor area, followed by gradual clearance over time (Figure 5A). 

The liver, spleen, lung, kidney, intestine, and tumor were excised on the last day to analyze antibody distribution in specific organs. Compared with the control human IgG, h4G3 showed a similar distribution in normal organs, whereas it accumulated significantly in tumor tissues (Figure 5B). These results suggest that the h4G3 is highly specific for CLDN3-expressing tumors, and it is, therefore, suitable for the detection of aberrantly expressed CLDN3 on tumor tissues. This indicates its potential value as a diagnosis and for CLDN3-targeted therapy for the treatment of cancer.

## 4. Discussion

Overexpression of CLDN3 has been reported in various malignant carcinomas, and CLDN3 has emerged as a potential cancer biomarker and therapeutic target because of its surface expression pattern associated with tumorigenesis [4,5,6,7,8]. Antibody screening using recombinant proteins is not suitable for antibody development against antigens with complex structures such as four-transmembrane domain proteins including CLDNs. In a structural binding study between C-CPE and ECL2 of CLDN4, various GST fusion recombinant proteins containing different domains of CLDN4 reveal that ECL2 with the downstream transmembrane domain of CLDN4 shows optimal binding activity to C-CPE, suggesting that ECL2 in the context of neighboring domains displays better conformation than a separated peptide such as only the ECL2 domain [56]. Therefore, it is crucial to choose a proper antigen for developing antibodies capable of recognizing an antigen on the cell membrane.

In this study, we generated a human mAb (h4G3) against CLDN3 through the phage display technique using native antigens, stably CLDN3 overexpressing cell lines, and CLDN3-embedded lipoparticles. Unlike other murine or chimeric antibodies against CLDN3 [57,58,59], h4G3 is a human mAb, which minimizes the immune response and improves safety and efficacy. The h4G3 could bind CLDN3 on the cell membrane, whereas it did not detect CLDN3 in an immunoblot or formaldehyde-fixed samples or recombinant CLDN3 protein (data not shown), indicating that h4G3 recognizes the native conformation of CLDN3. 

The CLDNs have fairly conserved extracellular loops [11] which regulate paracellular tightness and selective ion permeability [10]. To avoid an off-target effect in targeting CLDN3, antibody specificity is important. The h4G3 demonstrated high specificity for CLDN3 among phylogenetically close members. In addition, h4G3 recognized mCLDN3, in which ECL2, the h4G3 binding region, differs from the human sequence by only 1 aa. The binding affinity of h4G3 for mCLDN3 was lower than hCLDN3. Especially, the association rate constant during the initial step (k_a1_; A + B ↔ AB) and formation rate constant during the conformational change step (k_a2_; AB ↔ AB*) for mCLDN3 were lower than hCLDN3 in the “one-to-one two-state” fitting. In the “one-to-two” fitting, the second association rate constant (k_a2_; AB_1_+ B_2_ ↔ AB_1_B_2_) for mCLDN3 was also lower than hCLDN3. The region that differs in ECL2 between hCLDN3 (150V) and mCLDN3 (150L) has a strong hydrophobic area at the turn region among helices [60]. In another structural study of platelet endothelial cell adhesion molecule-1 (PECAM-1) polymorphisms, the difference in side-chain size and orientation between valine and leucine affects the conformational structure of neighboring residues and leads to change in domain direction [61]. Thus, the different sequences in ECL2 may alter the binding affinity of h4G3 between hCLDN3 and mCLDN3. Nevertheless, the affinity of h4G3 for mCLDN3 showed a sub-nanomolar affinity in the “one-to-two” model by the avidity effect. All these suggest that the efficacy and safety of h4G3 can be evaluated in a mouse model to assess the adverse effects of targeting CLDN3.

The CLDN3 is overexpressed in various carcinomas including breast, colorectal, gastric, pancreatic, prostate, and ovarian cancer [17,18,19,20]. The ADCC activity of h4G3 according to CLDN3 expression levels was examined in representative cancer cell lines using NK-92MI-CD16a cells. Although ADCC was dependent on CLDN3 expression, ADCC was lower in the human pancreatic cell line AsPC-1 than in the gastric cancer cell line SNU-216 which has lower CLDN3 expression. Jochems et al. [62] reported that AsPC-1 cells are more resistant to lysis from the natural killing of NK-92 cells expressing CD16a than other cell lines. Potential mechanisms underlying resistance to ADCC, such as low sensitivity to the perforin–granzyme system or impaired perforin binding to the target cell surface [63,64], need to be examined to overcome resistance in cancer treatment.

The CLDN3 is also expressed in human normal tissues. For example, high levels of CLDN3 are observed in colon, rectum, thyroid, and salivary, and some levels of CLDN3 express in pancreas, prostate, liver, and kidney [21,65]. Therefore, selective targeting of tumor tissues is necessary for the safety of CLDN3-targeted therapy in clinical application. Both CPE- and C-CPE-based cancer therapies have shown promising anti-tumor efficacy and provide proof of concept for CLDN3-targeted therapy [27,28,31,66]. In intact TJs, which form paired TJ strands, CLDNs are sterically inaccessible to CPE because the CPE binding residues of CLDNs are blocked [67]. The molecular weight of CPE is 35 kDa, and antibodies with a molecular weight of 150 kDa cannot easily access CLDNs in intact TJ strands. Among other antibodies targeting cell adhesion molecules, those targeting carcinoembryonic antigen (CEA) show good distinction between tumor and normal tissues, and radioactively labeled cytotoxic antibodies are well tolerated [68,69,70]. An antibody targeting epithelial cell adhesion molecule (EpCAM) shows limited in vivo accessibility in normal tissues [71]. Because CLDN3 is a component of TJs, its accessibility to antibodies may be much lower than that of CEA and EpCAM. The h4G3 conjugated to a fluorescent dye showed a lower distribution in normal tissues than in the tumor site in mice bearing xenograft tumors despite its ability to recognize mouse CLDN3. To assess the tissue cross-reactivity of h4G3, the immunohistochemistry of human normal tissues demonstrated that h4G3 mainly detected CLDN3 in glandular epithelium of gastrointestinal tract rather than smooth muscles, connective tissues or other organs (data not shown). These findings suggest that h4G3 could be applied to targeting CLDN3-positive cancer.

Although h4G3 accumulated in the tumor in mouse xenograft models, in vivo anti-tumor efficacy was not observed (Figure S9). The ADCC depends on several parameters such as antibody-FcγR binding affinity, density and stability of the antigen on the surface of the target cell, and antibody–antigen affinity. The hIgG1 is the most commonly used isotype as a therapeutic antibody because of its excellent efficacy, such as ADCC, by binding to the FcγRs on effector cells such as NK cells, macrophages, and dendritic cells. The hIgG1 can bind to all activating mFcγRs and induces ADCC in mouse NK cells and mouse macrophages. Although the activation of cellular immune effector function by hIgG1 is strong, the in vitro ADCC activity of an anti-EGFR hIgG1 is three times lower than that of mIgG1 using mNK cells, and hIgG1 is not as effective as mIgG2a in an A431 xenograft model [72]. Therefore, the function of hIgG1 might be underestimated in mouse models compared with humans. Regarding the antigen, CLDN3 is becoming accessible during epithelial tumorigenesis through misorientation of the cell division axis [7]. However, not all CLDN3 become accessible and some still maintain tight junctions. Thus, the density of the antigen could be too low to cause the effector function. Because of the complicated factors involved in the immune reaction, the efficacy of antibodies targeting CLDN3 may be diminished in mouse xenograft models. To date, previously known anti-CLDN3 antibodies have not shown in vivo anti-cancer efficacy [57,73]. The dual-targeting antibodies against CLDN3 and CLDN4 have shown anti-cancer efficacy in a preventive model in which the antibody was injected on day 0 after the inoculation of cancer cells [58,59]. However, in patients, cancer has already been formed and has a different microenvironment. Therefore, a definite therapeutic efficacy is needed.

**Figure 6:**
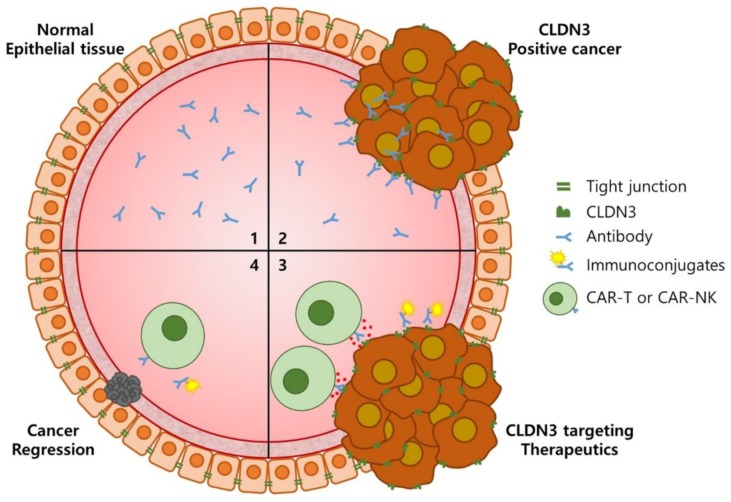
Cancer specificity of h4G3 and its applicability to cancer treatment. In normal epithelial tissues, h4G3 cannot access CLDN3 in tight junctions (**1**). When epithelial cells are transformed into cancer cells, the cancer cells proliferate through out-of-plane division, resulting in external exposure of CLDN3. Therefore, h4G3 specifically recognizes CLDN3-positive cancer cells (**2**). The h4G3 could, therefore, be applied to immunoconjugates or CAR immunotherapy for the treatment of CLDN3-positive cancer (**3**). Consequently, this may lead to the regression of CLDN3-positive cancer with few side effects (**4**). CAR, chimeric antigen receptor.

Recently, immunoconjugates have received attention as promising targeted therapies for cancer treatment. Antibodies that deliver drugs, such as pharmacologic agents, radioisotopes or photosensitive agents, to tumor cells can improve tumor-to-normal tissue selectivity and specificity in cancer treatment [47,74]. In nuclear medicine, antibodies are especially applied to theranostics which provide both diagnosis and therapy [75,76]. Furthermore, tumor immunotherapy by introducing a chimeric antigen receptor (CAR) into T cells or NK cells through genetic modification has been demonstrated as a promising strategy for cancer treatment [55,77,78]. The CAR-engineered immune cell therapy has shown good responses in hematological malignancies and achieved successful trials in solid tumors. Especially, CLDN18.2-specific CAR-T cells generated using scFv from a developed antibody show promising therapeutic efficacy in gastric cancer [79]. The h4G3-activated NK-92MI-CD16a cells function through a similar but indirect mechanism to that of CAR activation, indicating their potential application in CAR-NK cells. Altogether, the ability of h4G3 to specifically recognize CLDN3-positive tumor cells suggests that it could be applied to immunoconjugates and CAR immunotherapy (Figure 6). For these reasons, we are currently testing the efficacy of h4G3 conjugated to radioisotopes such as ^111^In or ^188^Re as well as nanoparticles containing polydopamine (PDA) which generate heat when irradiated with near-infrared laser. In addition, we plan to develop CAR-NK cells using the 4G3 scFv sequence against CLDN3-positive solid tumors.

In summary, scFv phage display using CLDN3-expressing CHO-K1 cells and CLDN3-embedded lipoparticles as antigens was used to generate the anti-CLDN3 human mAb (h4G3). We showed that h4G3 was highly specific for CLDN3 in vitro and in vivo. Furthermore, h4G3 may be suitable for cancer imaging, therapeutic agent conjugation, and CAR immunotherapy for CLDN3-positive cancer.

## 5. Conclusions

In conclusion, we developed human mAb (h4G3) against CLDN3 through scFv phage display using CLDN3-expressing CHO-K1 cells and CLDN3-embedded lipoparticles. The present approaches and results provide a good reference to generate mAbs against CLDNs or membrane proteins with complex structures. The h4G3 was highly specific for CLDN3—not for other CLDN family members—showed ADCC activity depending on CLDN3 expression, and preferentially accumulated in tumor tissues over normal tissues. Therefore, h4G3 is a promising candidate for conjugation to various imaging probes and therapeutic agents, and for CAR immunotherapy for the diagnosis and treatment of CLDN3-expressing pan-carcinoma.

## Figures and Tables

**Table 1:** Summary of binding kinetics fitted by “one-to-one two-state” model.

CLDN3 Cell Lines	k_a1_ (M^−1^s^−1^)	k_d1_ (s^−1^)	k_a2_ (s^−1^)	k_d2_ (s^−1^)	K_D_ (nM)
hCLDN3/HEK293	4.66 × 10^4^	6.71 × 10^−4^	1.56 × 10^−4^	4.35 × 10^−5^	4.03
hCLDN3/TOV-112D	4.74 × 10^4^	3.37 × 10^−4^	7.77 × 10^−4^	2.57 × 10^−5^	2.35
mCLDN3/HEK293	8.69 × 10^3^	7.07 × 10^−4^	8.63 × 10^−5^	2.27 × 10^−5^	20.4

**Table 2:** Summary of binding kinetics fitted by “one-to-two” model.

CLDN3 Cell Lines	k_a1_ (M^−1^s^−1^)	k_d1_ (s^−1^)	K_D1_ (nM)	k_a2_ (M^−1^s^−1^)	k_d2_ (s^−1^)	K_D2_ (nM)
hCLDN3/HEK293	3.57 × 10^4^	2.98 × 10^−4^	8.33	3.03 × 10^4^	1.53 × 10^−5^	0.504
hCLDN3/TOV-112D	6.66 × 10^4^	3.43 × 10^−4^	5.15	2.90 × 10^4^	1.26 × 10^−5^	0.434
mCLDN3/HEK293	2.42 × 10^4^	7.28 × 10^−4^	30.1	2.54 × 10^3^	1.91 × 10^−5^	7.54

## Supplementary Materials

The following are available online at https://www.mdpi.com/2218-273X/10/1/51/s1, Figure S1: Isolation of scFv against human CLDN3 by phage display. Figure S2: Generation of human monoclonal antibody (h4G3) from scFv against CLDN3. Figure S3: CLDN expression in stable CLDN transfectants. Figure S4: Comparison of CLDN3 level in various cancer cell lines. Figure S5: Fluorescence sensorgrams of h4G3–CLDN3 interaction on the cell membrane. Figure S6: Fluorescence-labeled and unlabeled h4G3 bind with equal strength. Figure S7: Generation of the CD16a-expressing NK-92MI cell line. Figure S8: In vitro cytotoxicity and CDC activity of h4G3. Figure S9: In vivo anti-tumor efficacy test of h4G3.
