# Supplementary material for: Development of Human Monoclonal Antibody for Claudin-3 Overexpressing Carcinoma Targeting
Source: Biomolecules. 2019 Dec 28;10(1):51. doi: 10.3390/biom10010051 (PMC7022679; doi:10.3390/biom10010051)
Supplement: Supplementary file 1 [file biomolecules-10-00051-s001.zip › biomolecules-640889-SI.pdf]

biomolecules

Supplementary Information

Development of human monoclonal antibody for Claudin-3 overexpressing carcinoma targeting

Hobin Yang[2], Hayeon Park[3], Yong Jin Lee[3], Jun Young Choi[3], TaeEun Kim[2], Nirmal Rajasekaran[2], Saehyung Lee[1], Kyoung Song[4], Sungyoul Hong[2], Joon-Seok Choi[5], Hyunbo Shim[6], Young-Deug Kim[3], Soohyun Hwang[7], Yoon-La Choi[7], Young Kee Shin[1,2,8,*]

[1] Molecular Medicine and Biopharmaceutical Sciences, Graduate School of Convergence Science and Technology, Seoul National University, Seoul, Korea

[2] Laboratory of Molecular Pathology and Cancer Genomics, Research Institute of Pharmaceutical Sciences and College of Pharmacy, Seoul National University, Seoul, Korea

[3] ABION Inc., R&D Center, Seoul 08394, Korea

[4] Center for Companion Diagnostics, LOGONE Bio Convergence Research Foundation, Seoul, Korea

[5] College of Pharmacy Daegu Catholic University Hayang-ro 13-13 Gyeongsan-si, Hayang-eup, Gyeongbuk 38430, Korea

[6] Department of Life Science, College of Natural Science, Ewha Womans University, Seoul, Korea

[7] Department of Pathology, Samsung Medical Center, Sungkyunkwan University School of Medicine, Seoul, Korea

[8] College of Pharmacy, Bio-MAX, and Research Institute of Pharmaceutical Sciences, Seoul National University, Seoul, Korea

* Correspondence: ykeeshin@snu.ac.kr

Materials and Methods

scFv phage display screening

A human single-chain Fv (scFv) phage library was used to develop a specific antibody against CLDN3. hCLDN3/CHO-K1 cells and hCLDN3-embedded lipoparticles (Integral Molecular Inc., Philadelphia, PA, USA) were selected as antigens because they represent the natural conformation of human CLDN3. For hCLDN3/CHO-K1 cell panning, the scFv library was blocked with 3% FBS in PBS and mixed with 1×10^7 CHO-K1 cells for 1 h to remove non-specific binding. The solution was centrifuged at 2,000 rpm for 2 min, and scFv in the supernatant was incubated with 1×10^7 hCLDN3/CHO-K1 cells for 1 h. Cells were centrifuged at 2,000 rpm for 2 min and washed with 3% FBS in PBS. To elute the bound scFv, 100 mM triethylamine (pH 10) was added, incubated for 5 min, and neutralized with 1 M Tris-HCl (pH 7.4). The isolated scFv was used to infect E. coli TG1, which was grown overnight at 37°C on LB agar plates containing ampicillin. Then, E. coli TG1 was harvested and incubated at 37°C on a shaker in SB (3% tryptophan, 2% yeast extract, and 1% MOPS) liquid medium containing ampicillin until reaching an OD600 of 0.5. E. coli TG1 was then infected with $1 \times 10^{11} – 1 \times 10^{12}$ helper phage (VCSM13) for 1 h and incubated overnight at 30°C on a shaker after kanamycin addition. For phage rescue, E. coli TG1 was centrifuged at 7,000 rpm for 15 min, and the supernatant was mixed with 5× PEG solution (20% PEG8000, 15% NaCl) with vigorous vortexing and then incubated for 30 min at 4°C. The phage pellet was collected by centrifugation at 15,000 rpm for 10 min and resuspended in PBS. Pellet insoluble matter was removed by centrifugation at 15,000 rpm for 10 min, and the supernatant was used for scFv phage library panning. The procedure was performed four times to amplify the specific scFv against human CLDN3. Panning with hCLDN3-embedded lipoparticles was performed in the same manner except that 4% skim milk was used for blocking and null lipoparticles were used for depletion. Phage output-to-input ratios were calculated for each round. A total of 190 clones randomly selected from the third and fourth round outputs were analyzed for scFv expression, antigen binding, and the scFv sequence. Primers used for scFv gene sequencing were as follows: 5′-AAGACAGCTATCGCGATTGCAG-3′ (forward), 5′-GCC CCCTTATTAGCGTTTGCCATC-3′ (reverse).

scFv ELISA binding assay

The CLDN3 binding ability of the scFv pool from each panning round as well as that of single scFv clones was evaluated by ELISA. scFv was induced by incubating E. coli with 1 mM isopropyl β-D-1-thiogalactopyranoside (IPTG) overnight at 30°C in a shaker. E. coli was lysed with TES buffer (200 mM Tris-HCl, 0.5 M sucrose, and 0.5 mM EDTA), and scFv was collected from the supernatant of the E. coli lysate by centrifugation at 3,000 rpm for 15 min. The scFv was incubated with 1×10^5 hCLDN3/L cells or four units of hCLDN3-embedded lipoparticles for 1 h. ELISA plates were then washed three times and incubated with 100 μL anti-HA-HRP (Santa Cruz Biotechnology, Santa Cruz, CA, USA) for 1 h. After three washes, TMB was added to generate a color reaction, and the absorbance was measured at 450 nm using a microplate reader.

Western blotting

The detailed procedure was listed in the materials and methods of main text. Primary antibodies used for western blotting were as follows: anti-CLDN3 (Invitrogen, Carlsbad, CA, USA), anti-CLDN5 (Abcam, Cambridge, UK), or β-actin (Santa Cruz Biotechnology).

Flow cytometry analysis

The detailed procedure was listed in the materials and methods of main text. Primary antibodies used for flow cytometry were as follows: anti-CLDN1-FITC (R&D Systems, Minneapolis, MN, USA), anti-CLDN3-FITC (R&D Systems), anti-CLDN4-FITC (R&D Systems), anti-CLDN6 (Abcam), anti-

CLDN8 (R&D Systems), anti-CLDN9 (Abcam), anti-CLDN17 (R&D Systems), or anti-CD16-FITC (Biolegend Inc., San Diego, CA, USA).

In vitro cytotoxicity and complement-dependent cytotoxicity (CDC) assay

Target cells were seeded on 96-well plates at a density of 2×10^4 cells/well and incubated overnight. After 24 h, the target cells were incubated with human IgG (Jackson Immunoresearch Laboratories, West Grove, PA, USA) or h4G3 at a final concentration of up to 10 μg/mL in a CO_2 incubator for 72 h at 37°C. The cytotoxicity was measured using WST assay. For CDC assay, the cells were incubated with various concentration of human IgG (Jackson Immunoresearch Laboratories) or h4G3 at a final concentration of up to 10 μg/mL in presence of 25% human serum (v/v) for 6 h. Viable cells were quantified using WST assay.

In vivo anti-tumor efficacy test in nude mice xenograft models

To generate xenograft, 5×10^6 OVCAR-3 cells in 100 μL PBS were injected subcutaneously into the right flank of 6-week-old female athymic nude mice (Orient Bio, Seongnam, Gyeonggi, South Korea). For MCF-7 xenograft model, 5×10^6 MCF7 cells in 100 μL PBS were injected subcutaneously into athymic nude mice planted with 17β-estradiol pellets (Innovative Research of America, Sarasota, FL, USA). After tumor size reached ≈200 mm^3, human IgG (Jackson Immunoresearch Laboratories) or h4G3 was injected intravenously at 10 mg/kg three times a week for 3 weeks. Tumor size was monitored twice a week. Tumor volume was calculated by the following equation: tumor volume = length × $width^2/2$.

Supplementary Figures

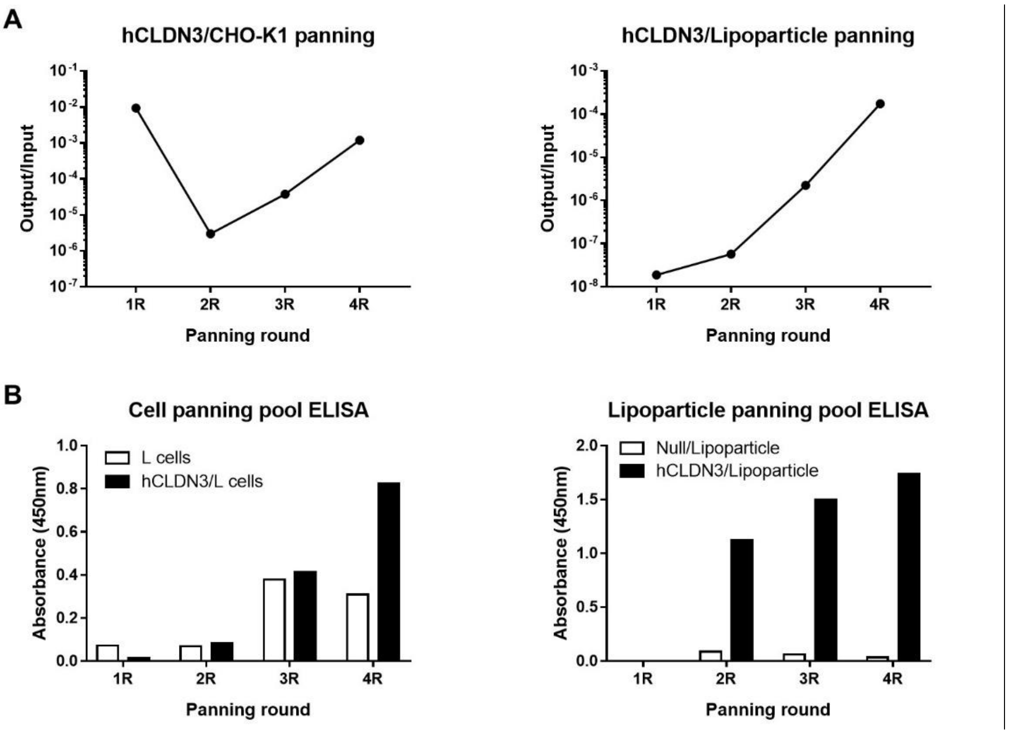

Figure S1. Isolation of scFv against human CLDN3 by phage display. (A) Phage output-to-input ratios from each panning round of scFv phage display on hCLDN3/CHO-K1 and hCLDN3-embedded lipoparticles were calculated and the ratio increased till the final panning round. (B) The phage pool from each round of panning was analyzed to confirm the amplification of scFv against CLDN3 by hCLDN3/L cells cell-based and hCLDN3/lipoparticle-based ELISA. The signals were adjusted by subtraction of the blank value.

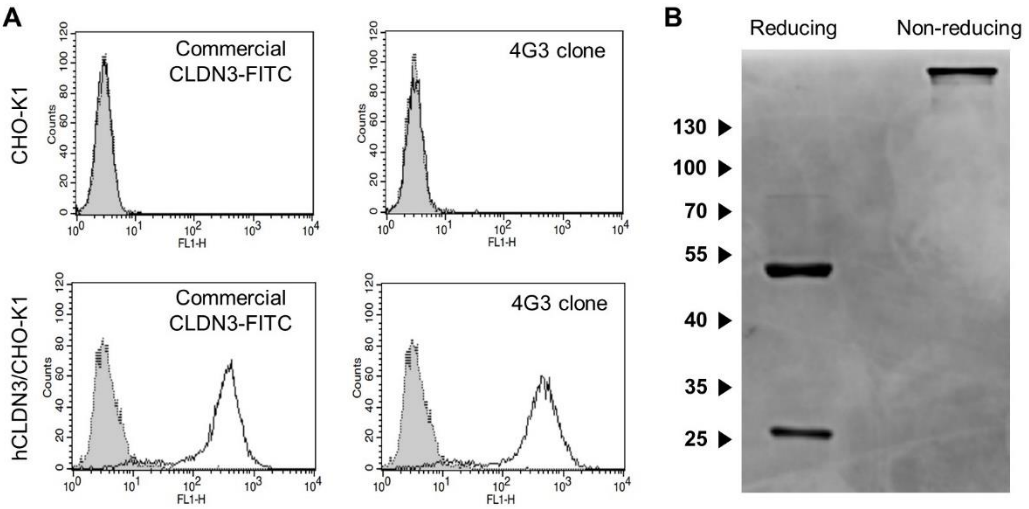

Figure S2. Generation of human monoclonal antibody (h4G3) from scFv against CLDN3. (A) To verify the binding specificity to CLDN3, 4G3 scFv clone was examined by flow cytometry using CHO-K1 cells and hCLDN3/CHO-K1 cells. Commercial CLDN3-FITC antibody was used as positive control. The gray closed dotted histogram represents FITC-conjugated anti-HA secondary antibody and the open solid histogram represents CLDN3-FITC and 4G3 clone. hCLDN3, human CLDN3. (B) CHO-S cells were stably transfected with an expression vector encoding h4G3. The antibody was purified from culture fluids using a Protein A column and separated by SDS-PAGE under reducing and non-reducing conditions.

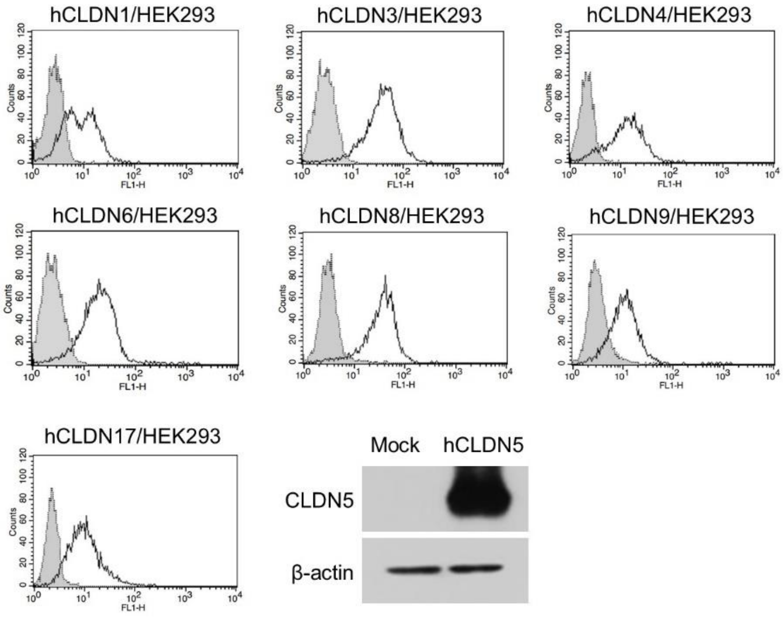

Figure S3. CLDN expression in stable CLDN transfectants. HEK293 cells stably expressing CLDNs were analyzed by flow cytometry and western blotting. For CLDN1, 3, and 4, FITC-conjugated commercial CLDN antibodies were incubated with stably transfected cells. The gray closed dotted histogram and the open solid histogram represent mock and antibody treated cells, respectively. For CLDN6, 8, 9, and 17, commercial CLDN antibodies were added to the stably transfected cells followed by FITC-conjugated secondary antibodies. The gray closed dotted histogram and the open solid histogram represent secondary antibody and primary antibody treated cells, respectively. For CLDN5, the expression of hCLDN5 was confirmed by western blotting at 23 kDa.

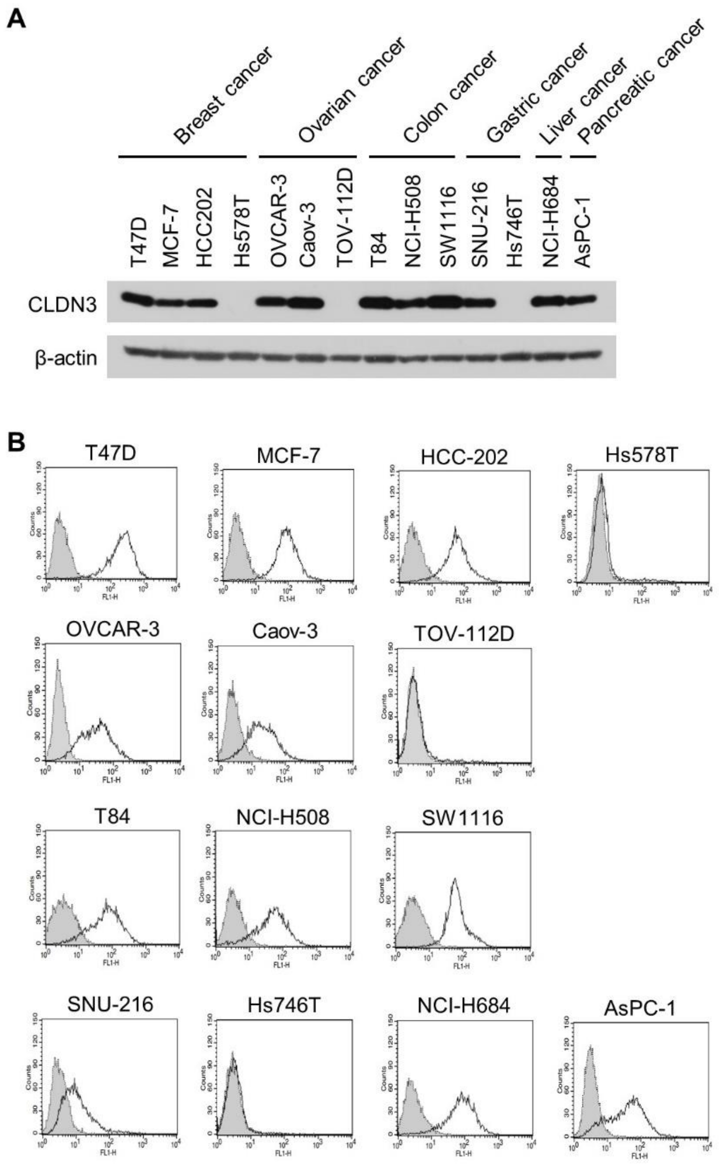

Figure S4. Comparison of CLDN3 level in various cancer cell lines. (A) Protein expression of CLDN3 was confirmed by western blotting using commercial CLDN3 antibody in various cancer cell lines. β-actin was used as the loading control. (B) Binding of h4G3 was assessed in various cancer cell lines by flow cytometry. Cell lines were incubated with h4G3 at 10 μg/mL, and then stained with FITC-conjugated goat anti-human IgG. The gray closed dotted histogram represents control human IgG and the open solid histogram represents h4G3.

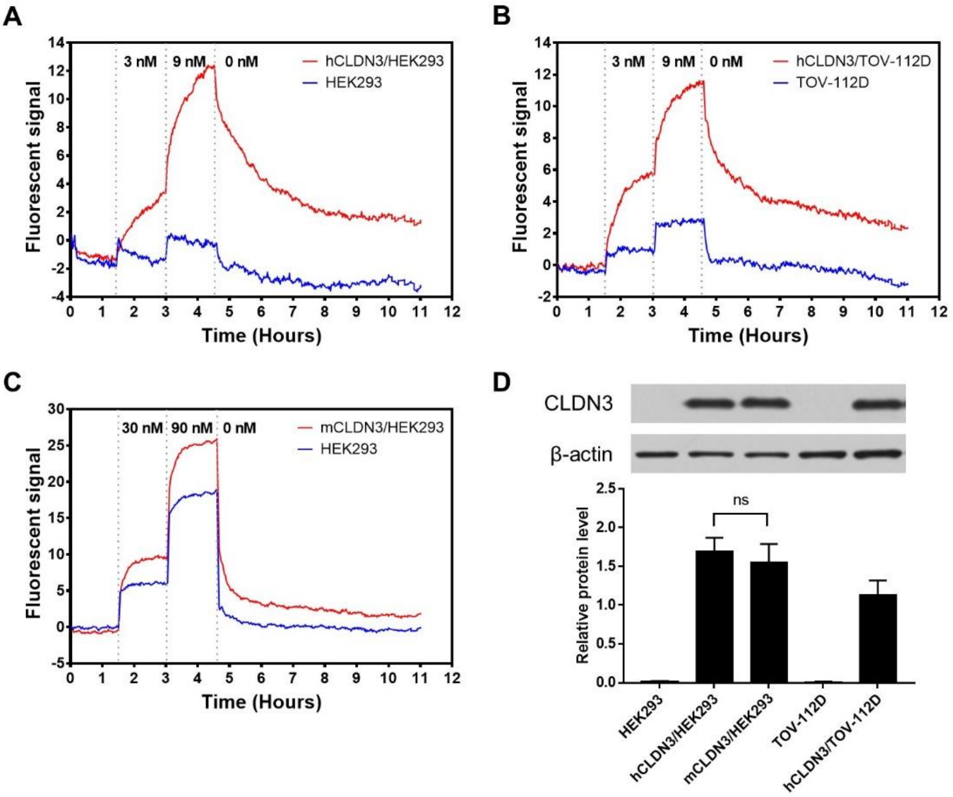

Figure S5. Fluorescence sensorgrams of h4G3-CLDN3 interaction on the cell membrane. FITC-labeled h4G3 was added stepwise to a final concentration of 3 nM (t = 1.5 h), 9 nM (t = 3 h) for hCLDN3 (A and B), and 30 nM (t = 1.5 h), 90 nM (t = 3 h) for the mCLDN3 (C). The dissociation phase was measured by replacement with fresh medium (t = 4.5 h). Fluorescent signals from positive and negative cell lines were recorded. (D) The expression level of CLDN3 was assessed by western blotting and normalized to that of β-actin in three independent experiments.

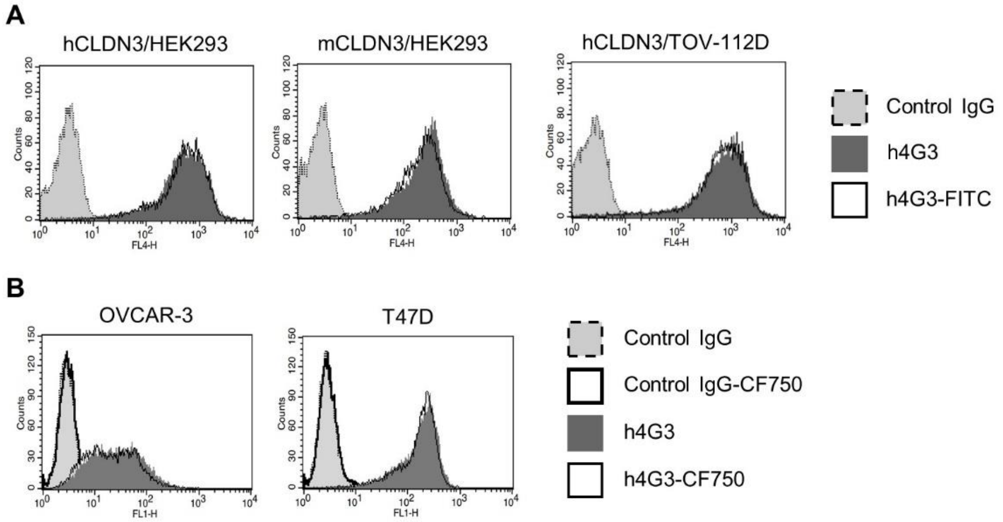

Figure S6. Fluorescence-labeled and unlabeled h4G3 bind with equal strength. (A) hCLDN3/HEK293, mCLDN3/HEK293, and hCLDN3/TOV-112D cells were incubated with FITC-labeled h4G3 used in binding kinetics and unlabeled h4G3. Bound antibodies were detected using anti-hIgG-Alexa647 secondary antibody and analyzed by flow cytometry. (B) OVCAR-3 and T47D cells were incubated with CF750-labeled h4G3 used in the xenograft model and unlabeled h4G3. Bound antibodies were detected using anti-hIgG-FITC secondary antibody and analyzed by flow cytometry.

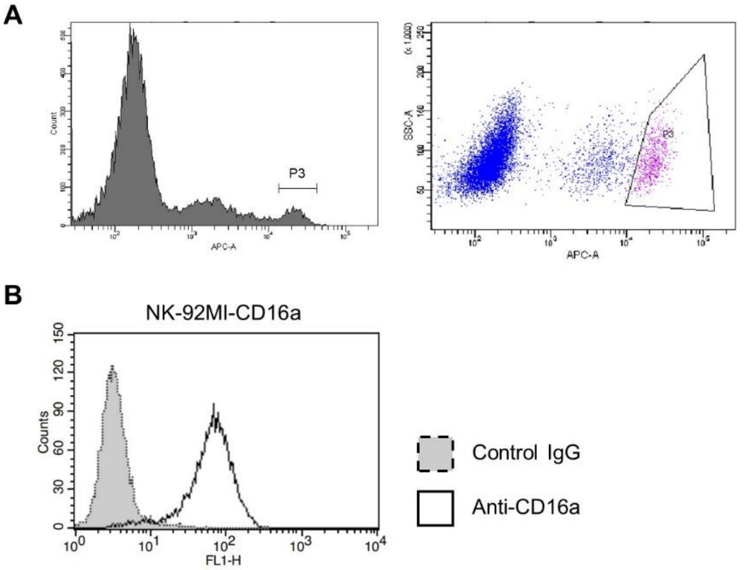

Figure S7. Generation of the CD16a-expressing NK-92MI cell line. (A) NK-92MI cells were transfected with CD16a by electrophoresis, and cells stably expressing CD16a were sorted. The cells were stained with APC-conjugated anti-CD16a antibody, and high expressing populations were gated and isolated using BD FACSAria™ III. (B) Isolated CD16a-expressing NK-92MI cells were incubated with Fc blocker and stained with FITC-conjugated anti-CD16a antibody. The gray closed dotted histogram and the open solid histogram represent FITC-conjugated control mouse IgG and FITC-conjugated anti-CD16a antibody, respectively.

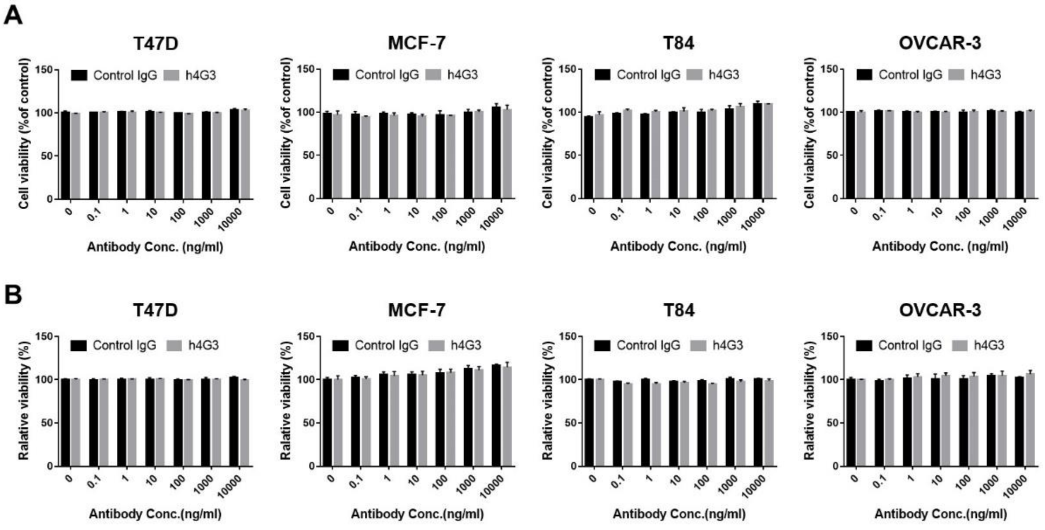

Figure S8. *In vitro* cytotoxicity and CDC activity of h4G3. (A) The cytotoxicity of h4G3 was determined in CLDN3 expressing cell lines. After 24 h of cell seeding on 96-well plate, the cells were treated with control IgG or h4G3 for 72 h at various concentrations. The cytotoxicity was measured using WST assay. (B) The CDC activity of h4G3 was determined in CLDN3-expressing cell lines. After 24 h of cell seeding on 96-well plate, the cells were incubated with various concentration of control IgG or h4G3 in presence of 25% human serum (v/v) for 6 h. Viable cells were quantified using WST assay. Data represent means ± SD (n=3).

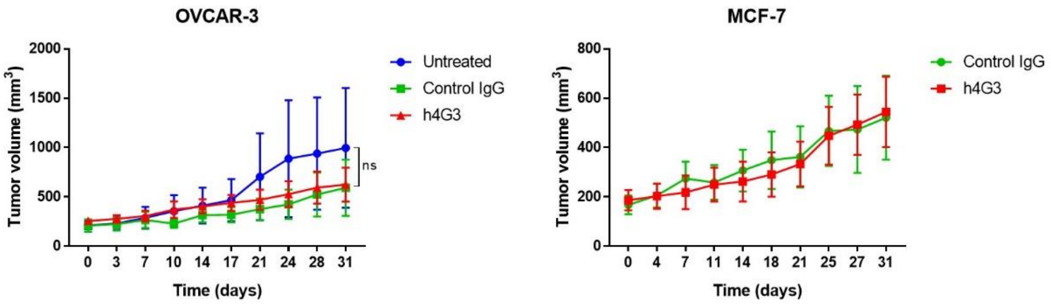

Figure S9. *In vivo* anti-tumor efficacy test of h4G3. OVCAR-3 and MCF-7 cells were transplanted subcutaneously into athymic nude mice. After tumor formation, h4G3 was intravenously injected at 10 mg/kg three times a week for 3 weeks. Tumor size was monitored twice a week. Tumor volume was calculated by the following equation: tumor volume = length × width^2/2. Data represent mean ± SEM (n=4).
